# Host–Microbe Interactions in Healthy and CSOM-Affected Middle Ears

**DOI:** 10.3390/microorganisms13020339

**Published:** 2025-02-05

**Authors:** Michel Neeff, Wandia Kimita, Sharon Waldvogel-Thurlow, Richard G. Douglas, Kristi Biswas

**Affiliations:** 1 Department of Paediatric Otolaryngology-Head and Neck Surgery, Starship Children’s Hospital, Te Whatu Ora, Health New Zealand, Te Toka Tumai, Auckland 1023, New Zealand; 2 Department of Surgery, The University of Auckland, Auckland 1023, New Zealandsharon.waldvogel-thurlow@auckland.ac.nz (S.W.-T.); richard.douglas@auckland.ac.nz (R.G.D.); 3 Department of Otolaryngology-Head and Neck Surgery, Te Whatu Ora, Health New Zealand, Te Toka Tumai, Auckland 1023, New Zealand

**Keywords:** *Staphylococcus aureus*, *Pseudomonas aeruginosa*, CSOM, cholesteatoma, microbes, host–microbe interactions

## Abstract

Chronic suppurative otitis media (CSOM) is a chronic middle ear inflammatory condition due to persistent polymicrobial middle ear infection. The interaction between local immune responses and microbial communities is not well understood, complicating the development of targeted therapies. This study aimed to characterise local immune cell responses and microbial composition in CSOM-affected middle ear mucosa, focusing on *Pseudomonas aeruginosa* and *Staphylococcus aureus*. A total of 24 CSOM patients and 22 controls undergoing tympanomastoid surgery participated in this prospective study. Middle ear and mastoid mucosa were collected for histological and microbiological analysis. Bacterial identification was performed using standard culture methods and Vitek MS, while immune cell populations were quantified via immunohistochemistry. Statistical analyses were performed using Kruskal–Wallis and Mann–Whitney tests. Microbiology results identified multiple pathogens in CSOM, including *S. aureus* and *P. aeruginosa*, with polymicrobial infections in 10 samples. CSOM patients exhibited significantly elevated immune cells, including CD3^+^, CD20^+^, and CD68^+^ cells, compared to controls. Histological analysis showed Gram-positive bacteria in three mastoid samples, with positive antibody staining for *S. aureus* (20.8%) and *P. aeruginosa* (12.5%) in CSOM patients. Controls had no bacterial staining. Intracellular bacteria may evade host defences and reduce antibiotic efficacy, contributing to CSOM persistence. Targeting intracellular pathogens in future treatments, along with studying polymicrobial communities, could improve management strategies.

## 1. Introduction

Chronic suppurative otitis media (CSOM) is a persistent inflammatory condition of the middle ear and mastoid cavity that presents with recurrent ear discharge lasting over six weeks through a perforated tympanic membrane [1,2,3]. It may occur with or without cholesteatoma, an abnormal accumulation of squamous epithelial cells that can extend into the middle ear or form epithelial inclusion cysts [4]. CSOM is a global health concern, contributing an estimated two million disability-adjusted life years (DALYs) annually [1,5,6]. It usually begins as acute otitis media (AOM), affecting up to 80% of children by age three, but in some cases, it progresses to CSOM, leading to chronic inflammation and persistent infection [7,8]. The pathogenesis of CSOM involves interactions between environmental factors, microbes, and host immune responses [5,8]. The chronicity and microbial persistence in CSOM complicate treatment, especially in resource-limited settings, and can lead to severe complications such as mastoiditis and meningitis, particularly in the presence of cholesteatoma [1,5]. Inflammatory mediators can damage sensory cells and disrupt the ossicular chain, leading to progressive hearing loss [9]. Symptoms such as otalgia, otorrhea, and ear pressure significantly impair quality of life, highlighting the need for a better understanding of CSOM’s underlying pathophysiology.

CSOM is often preceded by recurrent bacterial or viral AOM; if pathogens are not effectively cleared or if pro-resolution factors are deficient, chronic inflammation may ensue [10,11]. The resultant CSOM is characterised by sustained, potentially destructive interactions between host immunity and microbial pathogens [11,12,13], though the precise triggers for the transition from acute to chronic inflammation remain unclear. Immune responses involve CD4 and CD8 T cells which are elevated in the middle ear mucosa during infection [14], yet specific immune responses in diseased versus healthy tissue are not well characterised.

Research on the microbial pathogenesis of CSOM has primarily relied on culture-based techniques, identifying *Staphylococcus aureus* and *Pseudomonas aeruginosa* as the most frequently reported bacteria in patient swabs [15,16,17], including methicillin-resistant *S. aureus* (MRSA) [8,18]. Despite targeted antibiotic therapies, treatment remains largely ineffective [19,20]. Studies suggest that *P. aeruginosa* can exploit damaged epithelial barriers and produce toxins to evade host defences, contributing to persistent infections [21]. Factors such as biofilm formation and bacterial embedding in necrotic tissue (e.g., cholesteatoma), recurrent nasopharyngeal infections, and antibiotic resistance further complicate the pathology [22,23,24,25,26,27,28,29]. Notably, *S. aureus* biofilms have been identified in the middle ear mucosa of children with chronic otitis media [30], potentially contributing to CSOM by evading immune responses and antibiotic treatments [31,32,33].

Despite this knowledge, several gaps persist in the literature. The exact spatial distribution of pathogens within the epithelial layer of the middle ear mucosa and their interaction with host immune cells remains largely unexplored. Previous studies have focused predominantly on surface-level microbial colonization and biofilm formation [15,16,17], with limited investigations into deeper intramucosal microbial presence and host immune responses. Furthermore, there is insufficient understanding of how microbial localization within the tissue influences chronic inflammation, immune evasion, and treatment resistance. These gaps highlight the need for more refined approaches to investigate localized immune-microbial dynamics within the middle ear mucosa.

This study aims to address these gaps by investigating the colonization and spatial distribution of common clinically relevant pathogens, *P. aeruginosa* and *S. aureus*, along with local cellular immune responses in the middle ear mucosa of CSOM patients. Utilizing this approach, we combined histological techniques, including immunofluorescence and Gram staining, to simultaneously access the presence of pathogens and immune cells in the tissue of CSOM patients and a control group with healthy middle ear tissue.

## 2. Materials and Methods

### 2.1. Study Design and Ethics

This prospective study included 24 patients undergoing tympanomastoid surgery for CSOM with failed medical therapy, both with and without cholesteatoma, and 22 patients with healthy middle ears undergoing tympanomastoid surgery either for cochlear implantation or benign brain tumour (vestibular schwannoma) removal via the mastoid and middle ear. Middle ear and mastoid mucosa were collected by a single otolaryngologist at Auckland City Hospital, New Zealand. CSOM patients were categorized based on the presence or absence of cholesteatoma.

No patient had received antibiotics within two months of surgery, although all received intravenous cefazolin upon anesthesia induction in line with the institutional perioperative protocol. Patients with anatomical abnormalities of the temporal bone or immune deficiencies were excluded.

The study was approved by the New Zealand Health and Disability Ethics Committee (NTX/12/03/024), and written consent was obtained from all participants or their guardians prior to sampling.

### 2.2. Sample Collection

Tympanomastoid surgery was performed under general anesthesia and sterile conditions. Intraoperatively, mucosal samples from the middle ear and mastoid were harvested. Tissue samples, including mucosa and cholesteatoma, measuring approximately 2 to 5 mm were collected from the mastoid of CSOM patients. Mucosal samples measuring 2 to 3 mm were collected from the controls. A conventional microbiology swab was taken at the time of tissue collection. The tissue specimens were immediately fixed in Carnoy’s fixative (6:3:1 mixture of ethanol-chloroform-glacial acetic acid) for one week before being transferred to 70% ethanol and then embedded in paraffin.

### 2.3. Culture from Swabs

Conventional microbiology swabs from cholesteatoma or mucosa were sent to the hospital’s microbiology laboratory for routine bacterial culture. Swabs were inoculated onto Columbia Sheep Blood, Supplemented Chocolate with bacitracin, MacConkey, Colistin-Nalidixic, and Sabouraud Dextrose agar (Fort Richard Laboratories Ltd. 12 Huia Road, Auckland 1062, New Zealand). Columbia Sheep Blood and Supplemented Chocolate agar was incubated at 37 °C in ambient air with 5% CO_2_, while the other media were incubated at 37 °C in ambient air. Swabs from patients with chronic infections were also plated on Brain Heart Infusion media and incubated anaerobically. Bacterial species were identified using the Vitek^®^ MS Prime (bioMérieux, Marcy-l′Étoile, France) system, following Clinical and Laboratory Standards Institute (CLSI) guidelines. The growth of a single colony or more was considered positive.

### 2.4. Enumeration of Host Inflammatory Cells

**Figure 1:**
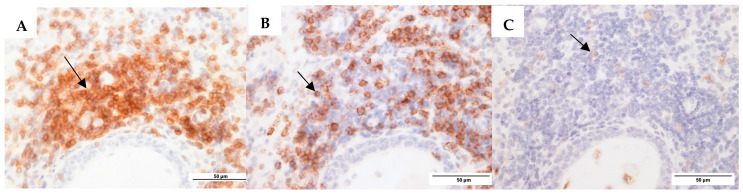
Representative images for mucosa CSOM. (**A**). CD3^+^ T cells (anti-CD3 monoclonal antibody (mAb)). (**B**). CD20^+^ B cells (anti-CD20 mAb). (**C**). CD68^+^ macrophages (anti-CD68 mAb). The black arrows are pointing to positively stained cells (brown colour) in each image.

Tissue sections (4 µm) were prepared from paraffin-embedded tissue, as described previously [34]. In brief, sections were processed separately for eosinophil and neutrophil cell counts using routine hematoxylin and eosin (H&E) staining; plasma cell counts using methyl green-pyronin staining; and CD3^+^ T cells, CD20^+^ B cells, and CD68^+^ macrophages using the respective anti-mAb, and they were visualized as seen in Figure 1. Immunohistochemistry was used to enumerate CD3^+^ T cells, CD20^+^ B cells, and CD68^+^ macrophages, following antigen retrieval using a pressurized heat-retrieval method (2100 retriever) with a citrate buffer (pH 6). Sections were incubated with either mouse anti-CD3 (IgG1), anti-CD20 (IgG2a), or anti-CD68 (IgG2a) (Leica Biosystems, Newcastle Upon Tyne, UK) and Novocastra IHC Diluent (Leica Biosystems, Newcastle Upon Tyne, UK) at the following dilutions: 1:400; 1:200; and 1:50. Sections were then processed with the Novolink Polymer Detection System Kit (Leica Biosystems, Newcastle Upon Tyne, UK) as per the manufacturer’s instructions. Negative controls (omitting the antibody) were included for all samples. Five representative high-power field images (63× magnification) were taken for each section using an epifluorescence Leica DMR upright microscope (Leica Microsystems, Wetzlar, Germany) with a SPOT camera (Diagnostic Instruments, Sterling Heights, MI, USA) and FIVE analysis software 5.0 (Olympus, Tokyo, Japan; Version 5.0). Cell counts were counted using ImageJ 1.54g (NIH, Bethesda, MD, USA; Version 1.54g), with replicates for each cell type averaged for analyses. Cell counts were conducted independently by two observers, with SW-T and MN blinded to subject diagnoses.

### 2.5. Gram Staining

Tissue sections (4 µm thick) were prepared from paraffin-embedded tissue. Prepared sections were processed separately for Gram staining, which was performed as per routine laboratory protocols. A subset of nine cases was Gram-stained for the middle ear and mastoid samples, and all remaining mastoid samples were also Gram-stained. Between 11 and 25 serial sections per case were screened using a Leitz camera (Leica) DMR upright microscope (Leica Microsystems, Wetzlar, Germany) with ×63 oil objective. A result was considered positive when there were Gram-positive cocci in the same location within the mucosa on three consecutive sections.

### 2.6. Immunofluorescence Staining for P. aeruginosa and S. aureus

Sections of tissue (4 μm) were blocked in normal goat serum for 30 min, followed by heat-induced epitope retrieval (HIER) in citrate buffers (10 mmol/L, pH 6) in the 2100 Retriever System (PickCell Laboratories, Amsterdam, The Netherlands) for the tissue that was stained with the S. aureus antibody. Sections were stained overnight at 4 °C with a monoclonal antibody for S. aureus clone staph 11-248.2 (1:500 dilution, MAB930, Merck Millipore, Temecula, CA, USA) and polyclonal antibodies for *P. aeruginosa* (1:1500 dilution, Abcam 74980 Cambridge, UK). The secondary antibody Goat anti-mouse (1:400 dilution, IgM 488, A21042 Invitrogen, Karlsbad, CA, USA) or Goat anti-chicken (1:400 dilution, IgY 594 Abcam, 150176 Cambridge, UK) antibodies were applied for 1 h at room temperature to fluorescently label bacteria. Then, 4′,6-diamidino-2-phenylindole (DAPI; Invitrogen, Carlsbad, CA, USA) was used to stain DNA. The sections were cover-slipped using Citifluor AF1 (Hatfield, PA, USA). Ten serial sections per case were stained with each antibody. These were visualized under fluorescence using a Leica DMR upright microscope (Leica Microsystems, Wetzlar, Germany) with a 100× oil objective. Photos were taken with the SPOT camera (Diagnostic Instruments, MI), and FIVE analysis software (Olympus, Tokyo, Japan; Version 5.0) was used to capture IHC fluorescent images. Image J (NIH, Bethesda, MD, USA; Version 1.54g) software was used to merge photos. A section was considered positive if the bacterial DNA was stained with DAPI, and this was surrounded by the antibody stain. A tonsil specimen containing either *S. aureus* or *P.* aeruginosa was used as a positive control.

A positive result for intracellular/interstitial bacteria was defined as having positive staining on three consecutive sections. A negative result was defined as having fewer than three positive stains. An uncertain result was defined as having non-consecutive positive staining on any three sections.

### 2.7. Data Analyses

Statistical differences between host immune cells were computed for the following groups: (1) disease state: CSOM versus controls; (2) anatomical location: mastoid versus middle ear; and (3) pathology: cholesteatoma versus non-cholesteatoma. Differences between groups were tested using the Kruskal–Wallis test or Mann–Whitney *t* test. Mastoid and middle ear samples were analysed separately for cholesteatoma and non-cholesteatoma patients. A *p* value of <0.05 was considered statistically significant.

## 3. Results

### 3.1. Participant Characteristics

A total of 46 patients were enrolled in the study, including 24 with CSOM and 22 controls. Among the controls, 14 underwent surgery for cochlear implants, while 7 underwent surgery for tumour removal. Of the CSOM patients, 16 had cholesteatoma and 8 had no cholesteatoma. There were no statistically significant differences in characteristics between the CSOM and control groups. Patient characteristics are summarized in Table 1.

### 3.2. Swab Culture Data

Culture data from conventional hospital microbiology swabs were negative for pathogens in the control group. In patients with CSOM, 6/14 middle ear samples and 5/15 mastoid samples from the cholesteatoma group were positive for pathogens. The non-cholesteatoma group had 4/8 positive middle ear cultures and 1/8 positive mastoid cultures. Identified microorganisms included *Propionibacterium* species, *P. aeruginosa*, *Klebsiella pneumoniae*, *Corynebacterium jeikeium*, *Actinomyces europaeus*, *Methicillin-resistant S. aureus* (MRSA), *Aspergillus flavus*, *Shewanella algae*, *Streptococcus constellatus*/*anginosus* group, *Parvimonas micra*, and various yeasts, skin flora, and anaerobes. Out of the specimens, 10 were polymicrobial and 4 were monomicrobial.

### 3.3. Differences in Host Immune Cells

#### 3.3.1. Comparison by Disease Status

The CSOM group exhibited a marked increase in CD3, CD20, CD68, lymphocytes, and histocytes compared to controls as shown in Table 2. In CSOM patients, CD3 levels (mastoid: median (IQR) 19.8 (3.1–86.3); middle ear: median (IQR) 6 (0.2–54.4)), CD20 (mastoid: median (IQR) 18.6 (0.4–52.3); middle ear: median (IQR) 17.8 (0–107)), and CD68 mastoid (median (IQR) 9 (2.4–23.5) and middle ear (median (IQR) 4.2 (1.4–9.9)), lymphocytes (mastoid: median (IQR) 44.7 (11–75.2); middle ear: median (IQR) 41 (10.4–67.2)), and histiocytes (mastoid: median (IQR) 24.3 (11.3–62.8); middle ear: median (IQR) 27.5 (3.0–61.2)) were significantly elevated (*p* < 0.01) compared to controls, where they were largely absent. Eosinophils, neutrophils, and plasma cells were present at low levels in CSOM and largely absent in controls, but the difference in cell counts was not statistically significant. Detailed results are shown in Table 2.

#### 3.3.2. Comparison by Anatomical Location

In CSOM, CD3, CD68, and CD20 numbers were slightly higher in the middle ear compared to the mastoid but this was not statistically significant. Lymphocytes, histiocytes, neutrophils, and plasma cells were comparable between both locations. Detailed results are shown in Table 2.

#### 3.3.3. Comparison Based on Pathology

There were no significant differences found between cholesteatoma and non-cholesteatoma samples for CD3, CD20, and CD68 cell counts. However, lymphocytes and histiocytes were elevated within each subgroup compared to neutrophils, eosinophils, and plasma cells, which is in keeping with a chronic, non-allergic immune response. Detailed results are shown in Table 2. Furthermore, no differences in immune cell profiles were observed in the control group, regardless of whether they underwent cochlear implantation or tumour removal (see Table S1 for data).

### 3.4. Histological Characterisation

Of the 24 patients undergoing mastoid surgery for CSOM and 22 patients with healthy middle ears undergoing either cochlear implantation (CI) or translabyrinthine surgery, 32 yielded sufficient mucosa to allow histological examinations.

#### 3.4.1. Gram and H&E Staining

**Figure 2:**
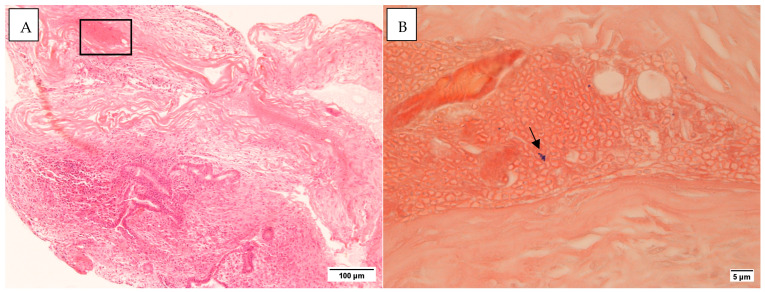
(**A**). H&E section ×10 (**B**). The area from the rectangle in (**A**) as seen in a Gram stain. The arrow points to Gram-positive cocci ×100. Taken on the Leica DMR microscope with Nikon Digital Sight DS-5Mc-U1 cooled colour camera.

All middle ear mucosal samples examined were negative for Gram staining. In three cases with mastoid tissue, Gram-positive bacteria were detected (Figure 2). No inflammatory cells were identified in the vicinity of these bacteria. Histology staining for control samples was negative (Figure S1).

#### 3.4.2. Immunohistochemistry: Antibody-Based *S. aureus* and *P. aeruginosa* Staining

**Figure 3:**
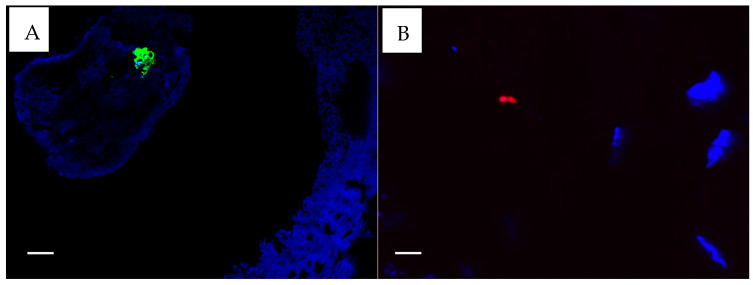
(**A**). *S. aureus* fluorescing green. (**B**). *P. aeruginosa* fluorescing red ×100. Taken with the FluoView FV1000 confocal microscope (Olympus, Auckland, New Zealand) using FluoView 4.2 software. The white scale bar on the images represent 5 µm.

All CSOM samples provided sufficient mucosa to allow antibody-based staining for *S. aureus* and *P. aeruginosa*. For five controls, insufficient material was available for *S. aureus* staining, and for nine controls, *P. aeruginosa* staining could not be performed due to insufficient mucosa. Only three CSOM subjects stained positive for *P. aeruginosa* (n = 24, 12.5%) (Figure 3B). Five CSOM subjects were positive for *S. aureus* (n = 24, 20.8%) (Figure 3A), and five were considered uncertain (n = 24, 21%). None of the controls had positive results for either stain, but two controls had uncertain positivity (n = 17, 12%) for *S. aureus* stains only. All bacteria identified at histological sections and with antibody-based staining appeared to be beneath epithelial surfaces, but whether they were located within cells could not be determined from the images.

## 4. Discussion

In this study, we investigated host–microbe interactions in the ear tissues of patients with CSOM using a novel approach that combined histological techniques, including immunofluorescence and IHC, and Gram staining to simultaneously access the presence of pathogens and immune cells in tissue biopsies. Our findings showed a significant increase in immune cell populations, including CD3 T cells, CD20 B cells, CD68 macrophages, lymphocytes, and histocytes, in CSOM tissue compared to the controls. This suggests a sustained chronic inflammatory response.

We identified multiple pathogens from swab culture data. These pathogens were absent in control samples, supporting the association of the polymicrobial nature of infection in patients with CSOM. This polymicrobial infection likely contributes to treatment resistance. Some organisms identified form biofilms, offering protection from host defences and antimicrobial agents [35]. Biofilm structures pose significant treatment challenges, reinforcing the need for therapies that target biofilm-associated pathogens [35]. While these findings are valuable, it is important to note that culture methods may underestimate microbial complexity. Our previous molecular studies revealed a more diverse bacterial community, highlighting the limitations of culture-based methods in identifying all potential pathogens [36,37].

Our data showed significantly higher levels of immune cells, including CD3 T cells, CD20 B cells, CD68 macrophages, lymphocytes, and histiocytes, in CSOM tissues compared to the controls. The anatomical continuity between the mastoid and middle ear was confirmed by similar immune cell numbers, suggesting consistent immune activity in both areas. There was sustained immune activation linked to chronic inflammation. Macrophages play a dual role, not only resolving inflammation but also contributing to chronic inflammation [38]. The recruitment of mononuclear leukocytes (monocytes and lymphocytes) in response to persistent pathogen exposure is in keeping with an inflammatory response aiming to contain infections [39,40]. Unlike acute infections, where pro-resolution mediators promote healing, chronic microbial exposure in CSOM prevents resolution, leading to continued immune cell recruitment and ongoing tissue injury and repair [41]. These findings align with previous studies that observed T-cell-mediated responses in CSOM patients [10,14,40].

Despite elevated immune cell levels in subjects, the numbers of eosinophils, neutrophils, and plasma cells did not significantly differ from controls. This suggests that CSOM triggers a localised immune response rather than a systemic one [42]. Unlike acute infections, which can induce systemic inflammatory markers, CSOM appears to provoke a chronic, non-allergic inflammatory response. This local immune activation may also facilitate immune evasion by pathogens, allowing them to persist without triggering a systemic inflammatory response.

Histological analysis revealed Gram-positive bacteria in some mastoid tissues, but not in middle ear samples or control tissues. The absence of inflammatory cells near bacteria may suggest that bacteria may evade immune detection or provoke minimal immune cell recruitment, enabling them to persist without eliciting strong inflammatory responses. While previous studies using culture and molecular techniques have reported bacterial presence in CSOM tissues [23,43], antibody-based staining in this study detected intramucosal *S. aureus* in 21% and *P. aeruginosa* in 13% of CSOM cases, lower than culture-based reports [15,16,17]. This may indicate immune evasion by pathogens. Small colony variants, particularly with respect to *S. aureus* and *P. aeruginosa*, may contribute to CSOM virulence, as they are adept at evading immune responses and antibiotic activity, form biofilms, and survive in host cells, all of which would support the chronicity and treatment resistance often seen in CSOM [44,45]. Such microcolonies are also seen in other chronic diseases, including chronic rhinosinusitis [46].

Although our primary focus was on *P. aeruginosa* and *S. aureus,* other bacteria, particularly anaerobes, may play a role in CSOM and warrant further investigation. The Gram-positive cocci observed in the Gram stain were presumed to be *S. aureus*, although other morphologically similar bacteria could be involved. Gram-positive anaerobic cocci (GPAC), known to contribute to polymicrobial soft tissue infections, have been identified in CSOM, with *Anaerococcuss* spp. and *Peptoniphilus* spp. found to dominate in cases involving cholesteatoma [37]. Additionally, *Alloiococcus otitidis,* commonly cultured from the external ear canal and considered part of the normal flora, may play a pathogenic role, as some studies have identified local antibody responses against *A. otitidis* in middle ear effusions from otitis media cases [47,48].

Bacterial invasion in ear tissue may likely occur through a disrupted epithelial barrier, allowing pathogens to bypass immune responses and reinfect the host [49]. In healthy mucosa, mechanisms such as epithelial layers, mucus, immunoglobulin A, and immune cells prevent bacterial invasion, as demonstrated by the absence of mucosal bacteria in control samples [50].

This study has several limitations. First, the small sample size and limited availability of normal mucosal specimens restrict the generalizability of the results. A larger cohort may reveal additional variability in immune and microbial profiles. Second, we did not assess the viability of intramucosal bacteria, limiting our understanding of the active roles of these microbes. Third, biofilms, known to contribute to pathogen persistence and treatment resistance, were not directly evaluated. Future studies with a larger sample size, viability assessments, and direct biofilm analysis could provide a more comprehensive understanding of pathogen behaviour and immune interactions in CSOM. Fourth, the inability to establish causality, specifically whether changes in immune cells are directly caused by pathogen presence, is outside the scope of this research. Future studies could use in vitro or ex vivo models with tissue biopsies to evaluate whether inflammatory cell populations change in response to pathogen exposure.

## 5. Conclusions

The persistent intracellular bacteria observed in our study may evade host defences and show reduced susceptibility to systemic antibiotics, potentially contributing to the recurrent nature of CSOM. This study provides findings which potentially contribute to the chronicity of CSOM. If these findings are confirmed in larger studies, therapies targeting bacteria that evade the immune system may become part of the treatment approach in CSOM. Further research on intracellular bacteria and polymicrobial communities, including GPAC, may provide valuable insights into the chronicity and treatment resistance of CSOM.

## Figures and Tables

**Table 1:** Characteristics of participants.

Characteristic	Controls (*n* = 22)	CSOM (*n* = 24)
Female, n (%)	7 (33)	8 (33)
Age, mean (SD)	33 (29)	33 (26.3)
Ethnicity		
European	13	9
Asian	9	5
Polynesian and Māori	3	10
Other (African)	1	0

Kruskal-Wallis test showed no difference between CSOM patients and controls. Abbreviation: SD—standard deviation.

**Table 2:** Differences in host immune cells of mastoid and middle ear of controls vs. CSOM patients. The values in the table are shown as median (interquartile range).

Host Immune Cells	Controls (*n* = 15)	CSOM (*n* = 24)	CSOM	
			**Cholesteatoma** **(*n* = 16)**	**Non-Cholesteatoma** **(*n* = 8)**
*Mastoid*				
CD3 T cells	0 (0–0)	19.8 (3.1–86.3) *	12.6 (3.1–72.9)	46.9 (13.1–88.9)
CD20 B cells	0 (0–0)	18.6 (0.4–52.3) *	18.6 (0.4–52)	20.7 (1.5–72.4)
CD68 macrophages	0 (0–0)	9 (2.4–23.5) *	19.8 (3.9–35.5)	7.4 (0.35–13.8)
Lymphocytes	0 (0–0)	44.7 (11–75.2) *	44.7 (10.8–74.8)	54.4 (21–78.4)
Histiocytes	0 (0–0)	24.3 (11.3–62.8) *	31.3 (12.2–67.2)	22.8 (11–61.2)
Eosinophils	0 (0–0)	0 (0–0.3)	0 (0–0)	0.3 (0–0.6)
Neutrophils	0 (0–0)	0.3 (0–1.3)	0.3 (0.2–1.1)	0.3 (0–3)
Plasma cells	0 (0–0)	0.6 (0–1.3)	0.7 (0–1.3)	0.3 (0–1.3)
*Middle ear*				
CD3 T cells	0 (0–0)	6 (0.2–54.4) *	18.2 (0.7–52.2)	4.2 (1.2–93.1)
CD20 B cells	0 (0–0)	17.8 (0–107) *	18 (3.1–97.3)	6.4 (0–130)
CD68 macrophages	0 (0–0)	4.2 (1.4–9.9) *	4.6 (2.1–9.8)	3 (0.8–8)
Lymphocytes	0 (0–0)	41 (10.4–67.2) *	50.4 (11.4–66.5)	30 (14.6–60.2)
Histiocytes	0 (0–0)	27.5 (3.0–61.2) *	27 (4.5–46.5)	28 (4.3–67.4)
Eosinophils	0 (0–0)	0 (0–0)	1 (0–0)	2 (0–0)
Neutrophils	0 (0–0)	0.3 (0–3.0)	0.3 (0–3.0)	0.8 (0.2–2.2)
Plasma cells	0 (0–0)	0.5 (0–2.0)	0.3 (0–1.9)	0.7 (0.4–4)

* Shows statistically significant difference (*p* < 0.01) between controls and CSOM patients. *p*-value generated using Mann–Whitney test. Abbreviation: CSOM—chronic suppurative otitis media.

## Data Availability

The data presented in this study are available on request from the corresponding author due to protecting the privacy of the children participating in this study.

## Supplementary Materials

The following supporting information can be downloaded at https://www.mdpi.com/article/10.3390/microorganisms13020339/s1, Table S1: Host immune cell count of mastoid and middle ear of CSOM and controls patients.; Figure S1: Ear mucosa sections from a CSOM patient that was IHC stained with (A) CD3, (B) CD20, (C) CD68 and (D) negative control, captured with a x5 magnification lens.
