# Supplementary material for: Host–Microbe Interactions in Healthy and CSOM-Affected Middle Ears
Source: Microorganisms. 2025 Feb 5;13(2):339. doi: 10.3390/microorganisms13020339 (PMC11858293; doi:10.3390/microorganisms13020339)
Supplement: Supplementary file 1 [file microorganisms-13-00339-s001.zip › microorganisms-3386309-supplementary.pdf]

Supplementary Material

Table S1. Host immune cell count of mastoid and middle ear of CSOM and controls patients.

Group		Patient ID	CD3 T cells	CD20 B cells	CD68 macrophages	lymphocytes	histiocytes	eosiniphils	neutrophils	plasma cells
**CSOM**	**Mastoid**	CWD_NC_1	17	2	0.4	6	65	1	0	0
		CWD_NC_2	59.2	35.8	14.4	37	60	0	3	0
		CWD_NC_3	1.4	0	0.2	26	74	0	0	0
		CWD_NC_4	34.6	5.6	8.4	75.4	24.3	0	0.3	0
		CWD_NC_5	137.8	50.8	6.4	89.6	10	0.3	0	0
		CWU_NC_1	89.4	137.2	31.2	71.7	21.3	0	3.7	3.3
		CWU_NC_2	0	0	0	0	0	0	0	0
		CWU_NC_3	88.8	259.8	13.6	87.3	11.3	0.6	0	0.6
		CWD_Ch_1	83.8	85.8	19.8	75	21.3	0.3	1.3	2
		CWD_Ch_2	8.4	0.8	7.4	0	0	0	0	0
		CWD_Ch_3	1.4	2.8	5	5	94.3	0	0	0.7
		CWD_Ch_4	10.4	8.4	68.6	16	81	0	1	2
		CWD_Ch_5	62	38.8	2.8	98.6	1	0	0.3	0
		CWD_Ch_6	0	0	47.4	74.6	24	0		1.3
		CWU_Ch_1	282.8	314.2	20.6	88.3	11.3	0	0.3	0
		CWU_Ch_2	0	0	0	0	0	0	0	0
		CWU_Ch_3	121.8	53.8	85.2	66	31.3	0	2.7	0
		CWU_Ch_4	0	0	0	0	0	0	0	0
		CWU_Ch_5	0	0	1.6	5.7	94.3	0	0	0
		CWU_Ch_6	100.6	198.8	23.6	86	13	0	0	1
		CWU_Ch_7	19.8	40.4	23.4	64.7	35.3	0	0	0
		CWU_Ch_8	22.4	0	9	25	73.6	0	0	1.3
		CWU_Ch_9	12.6	50.2	65.8	44.7	54.7	0	0.3	0.3
		CWU_Ch_10	4.8	18.6	2	39.3	60.7	0	0	0
	**Middle Ear**	CWD_NC_1	4.2	0	5	9	28	0	0	0
		CWD_NC_2	4.2	0	1.2	30	69.6	0	0.3	0
		CWD_NC_3	0	0	0	0	0	0	0	0
		CWD_NC_4	0.2	0	0.4	34	65.3	0	0	0.7
		CWD_NC_5	0	0	0	0	0	0	0	0
		CWU_NC_1	122.8	228.4	3	93	7	0	0	0

		CWU_NC_2	0	12.8	11	20.3	78.3	0	1.3	0
		CWU_NC_3	182.6	169	26	86.3	1.6	0	4.6	7.3
		CWD_Ch_1	0	0	0	0	0	0	0	0
		CWD_Ch_2	na	na	na	n/a	n/a	n/a	n/a	n/a
		CWD_Ch_3	75.8	193.4	9.6	68	19.4	0	3.3	9.3
		CWD_Ch_4	26.8	137.2	37.4	65	27	0.3	6	1.7
		CWD_Ch_5	54.4	306.4	10	98.6	1.3	0	0	0
		CWD_Ch_6	1.2	0.8	26.4	8.3	89.6	0	2	0
		CWU_Ch_1	100.4	106.8	2.2	90.3	9.3	0	0.3	0
		CWU_Ch_2	0.2	0	0	0	0	0	0	0
		CWU_Ch_3	19	23.2	3.8	63.7	36.3	0	0	0
		CWU_Ch_4	0	0	0	0	0	0	0	0
		CWU_Ch_5	2.8	18	2	65	35	0	0	0
		CWU_Ch_6	18.2	87.8	5.2	48	44	0	0	8
		CWU_Ch_7	0	8	6	18.7	81.3	0	0	0
		CWU_Ch_8	50	5.4	14.4	14.6	84	0	0	1.3
		CWU_Ch_9	6	17.8	2.2	50.4	49	0	0.3	0.3
		CWU_Ch_10	79.8	29.6	4.6	81.7	7.6	0	8.7	2
**Controls**	**Mastoid**	C_Cl_1	n/a	n/a	n/a	n/a	n/a	n/a	n/a	n/a
		C_Cl_2	n/a	n/a	n/a	n/a	n/a	n/a	n/a	n/a
		C_Cl_3	0	0	0	0	0	0	0	0
		C_Cl_4	0	0	0	0	0	0	0	0
		C_Cl_5	n/a	n/a	n/a	n/a	n/a	n/a	n/a	n/a
		C_Cl_6	n/a	n/a	n/a	n/a	n/a	n/a	n/a	n/a
		C_Cl_7	0	0	0	0	0	0	0	0
		C_Cl_8	0	0	0	0	0	0	0	0
		C_Cl_9	0	0	0	0	0	0	0	0
		C_Cl_10	0	0	0	7	93	0	0	0
		C_Cl_11	0	0	0	0	0	0	0	0
		C_Cl_12	n/a	n/a	n/a	n/a	n/a	n/a	n/a	n/a
		C_Cl_13	0.4	0	0.2	8.5	90.5	0	0.5	0.5
		C_Cl_14	0	0	0	0	0	0	0	0
		C_Cl_15	n/a	n/a	n/a	n/a	n/a	n/a	n/a	n/a
		C_T_1	0	0	0	0	0	0	0	0
		C_T_2	n/a	n/a	n/a	n/a	n/a	n/a	n/a	n/a
		C_T_3	n/a	n/a	n/a	n/a	n/a	n/a	n/a	n/a
		C_T_4	0	0	0	0	0	0	0	0
		C_T_5	0	0	0	0	0	0	0	0

	C_T_6	0	0	0	0	0	0	0	0
	C_T_7	0.6	0	0	16	45	0	4	0
**Middle Ear**	C_CI_1	n/a	n/a	n/a	n/a	n/a	n/a	n/a	n/a
	C_CI_2	n/a	n/a	n/a	n/a	n/a	n/a	n/a	n/a
	C_CI_3	0	0	0	0	0	0	0	0
	C_CI_4	n/a	n/a	n/a	n/a	n/a	n/a	n/a	n/a
	C_CI_5	0	0	0	0	0	0	0	0
	C_CI_6	n/a	n/a	n/a	n/a	n/a	n/a	n/a	n/a
	C_CI_7	0	0	0	0	0	0	0	0
	C_CI_8	0	0	0	0	0	0	0	0
	C_CI_9	0	0	0	0	0	0	0	0
	C_CI_10	n/a	n/a	n/a	n/a	n/a	n/a	n/a	n/a
	C_CI_11	0.2	0	0	0	0	0	0	0
	C_CI_12	0	0	0	0	0	0	0	0
	C_CI_13	0	0	0	0	0	0	9	0
	C_CI_14	0	0	0	0	0	0	0	0
	C_CI_15	n/a	n/a	n/a	n/a	n/a	n/a	n/a	n/a
	C_T_1	n/a	n/a	n/a	n/a	n/a	n/a	n/a	n/a
	C_T_2	1.2	0	0.4	0	0	0	0	0
	C_T_3	0	0	0	0	0	0	0	0
	C_T_4	n/a	n/a	n/a	n/a	n/a	n/a	n/a	n/a
	C_T_5	n/a	n/a	n/a	n/a	n/a	n/a	n/a	n/a
	C_T_6	0	0	0	0	0	0	0	0
	C_T_7	0	0	0	0	0	0	0	0

In CSOM patients Ch=cholesteatoma. NC = is non cholesteatoma.

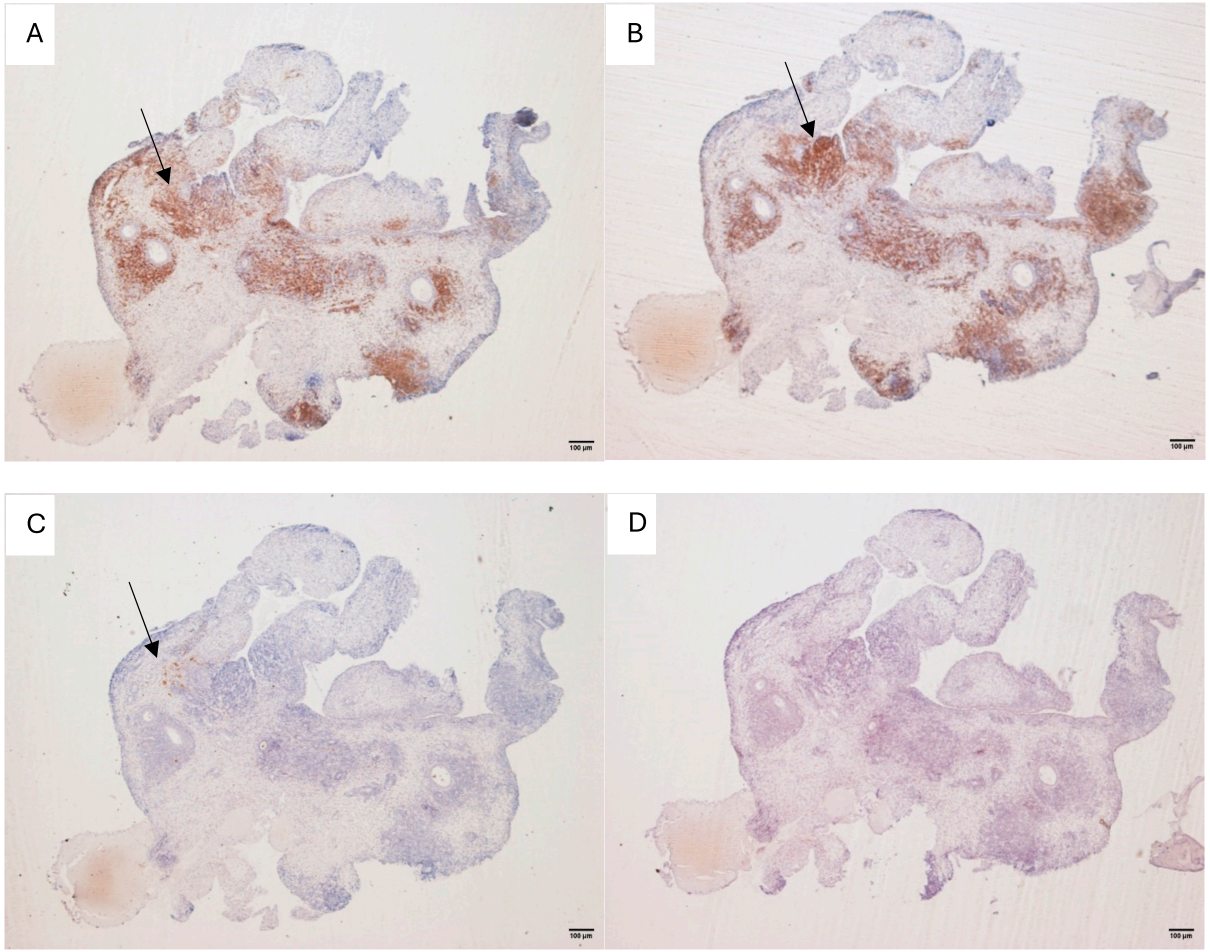

Figure S1 Ear mucosa sections from a CSOM patient that was IHC stained with (A) CD3, (B) CD20, (C) CD68 and (D) negative control, captured with a x5 magnification lens. The black arrow points to the IHC stained cells (brown staining) in each section. There is a lack of brown staining in the negative control. Images were captured on the Leica DMR microscope with Nikon Digital Sight DS-5Mc-U1 cooled colour camera.
